# Radiology AI Lab: Evaluation of Radiology Applications with Clinical End-Users

**DOI:** 10.1007/s10278-025-01453-2

**Published:** 2025-03-17

**Authors:** Olivier Paalvast, Merlijn Sevenster, Omar Hertgers, Hubrecht de Bliek, Victor Wijn, Vincent Buil, Jaap Knoester, Sandra Vosbergen, Hildo Lamb

**Affiliations:** 1 Leiden University Medical Center (LUMC), Leiden, the Netherlands; 2 Royal Philips B.V., Amsterdam, the Netherlands

**Keywords:** Radiology AI lab, Objective real-time sensing, Eye-tracking, User-state sensing

## Abstract

Despite the approval of over 200 artificial intelligence (AI) applications for radiology in the European Union, widespread adoption in clinical practice remains limited. Current assessments of AI applications often rely on post-hoc evaluations, lacking the granularity to capture real-time radiologist-AI interactions. The purpose of the study is to realise the Radiology AI lab for real-time, objective measurement of the impact of AI applications on radiologists’ workflows. We proposed the user-state sensing framework (USSF) to structure the sensing of radiologist-AI interactions in terms of personal, interactional, and contextual states. Guided by the USSF, a lab was established using three non-invasive biometric measurement techniques: eye-tracking, heart rate monitoring, and facial expression analysis. We conducted a pilot test with four radiologists of varying experience levels, who read ultra-low-dose (ULD) CT cases in (1) standard PACS and (2) manually annotated (to mimic AI) PACS workflows. Interpretation time, eye-tracking metrics, heart rate variability (HRV), and facial expressions were recorded and analysed. The Radiology AI lab was successfully realised as an initial physical iteration of the USSF at a tertiary referral centre. Radiologists participating in the pilot test read 32 ULDCT cases (mean age, 52 years ± 23 (SD); 17 male; 16 cases with abnormalities). Cases were read on average in 4.1 ± 2.2 min (standard PACS) and 3.9 ± 1.9 min (AI-annotated PACS), with no significant difference (*p* = 0.48). Three out of four radiologists showed significant shifts (*p* < 0.02) in eye-tracking metrics, including saccade duration, saccade quantity, fixation duration, fixation quantity, and pupil diameter, when using the AI-annotated workflow. These changes align with prior findings linking such metrics to increased competency and reduced cognitive load, suggesting a more efficient visual search strategy in AI-assisted interpretation. Although HRV metrics did not correlate with experience, when combined with facial expression analysis, they helped identify key moments during the pilot test. The Radiology AI lab was successfully realised, implementing personal, interactional, and contextual states of the user-state sensing framework, enabling objective analysis of radiologists’ workflows, and effectively capturing relevant biometrics. Future work will focus on expanding sensing of the contextual state of the user-state sensing framework, refining baseline determination, and continuing investigation of AI-enabled tools in radiology workflows.

## Background

Over 200 commercial artificial intelligence (AI) tools for radiology have been approved for use in the European Union [1], with the total doubling since 2021 [2]. Despite growing research interest and increasing industry investments, the anticipated widespread adoption of AI-drive workflow enhancements has yet to materialise] [2–5]. Many radiologists report that AI tools have not significantly reduced their practical workload [2–5].

Reports are emerging that an inadequate understanding of the impact of AI tools on the radiologist’s workflow may be partly responsible for the lacking adoption [6, 7]. Current assessments of AI implementation have primarily relied on post-hoc evaluations, such as surveys and questionnaires [8, 9]. While these methods provide valuable insights into user experience, they often lack the granularity required to capture the real-time interaction between radiologist and AI application. Consequently, important aspects such as workflow integration, decision-making processes, and user satisfaction may be overlooked.

To address these gaps, we introduce the user-state sensing framework (USSF), which emphasises real-time measurement of radiologist’s interactions with AI tools to provide a more comprehensive understanding of their impact on clinical workflows. We present the initial iteration of the Radiology AI lab, designed to simulate real-world radiology environments while enabling controlled evaluations of AI systems based on this framework. Additionally, we share findings from a pilot test conducted within the lab, aimed at assessing the baseline feasibility of real-time user-technology interaction measurement. Together, these results are essential before introducing AI-enabled systems for evaluation in future studies.

## Methods

### User-State Sensing to Support User-Technology Interaction Research

Traditional user-interaction research methods, such as observation, think-aloud protocols, and questionnaires, have long been used to evaluate system usability and workflow integration [10]. Advancements in sensor and computation technology now offer opportunities to complement the traditional approaches by capturing more specific, detailed, and real-time data on user interactions, including their engagement with AI systems [11]. For example, sensing may reveal insights if and how AI-marked findings have been noticed, evaluated, and responded to by a radiologist, and how this may be influenced by user characteristics (e.g. expertise level, fatigue), the task (e.g. case complexity), and the environment (e.g. causes of distraction).

Building on exploratory academic, technology, and solution provider research, we propose the user-state sensing framework. This framework is intended to structure the many sensing possibilities in a logical framework for discussion, research, and implementation in lab contexts and is not necessarily complete, ambiguous, and/or exhaustive.

**Fig. 1:**
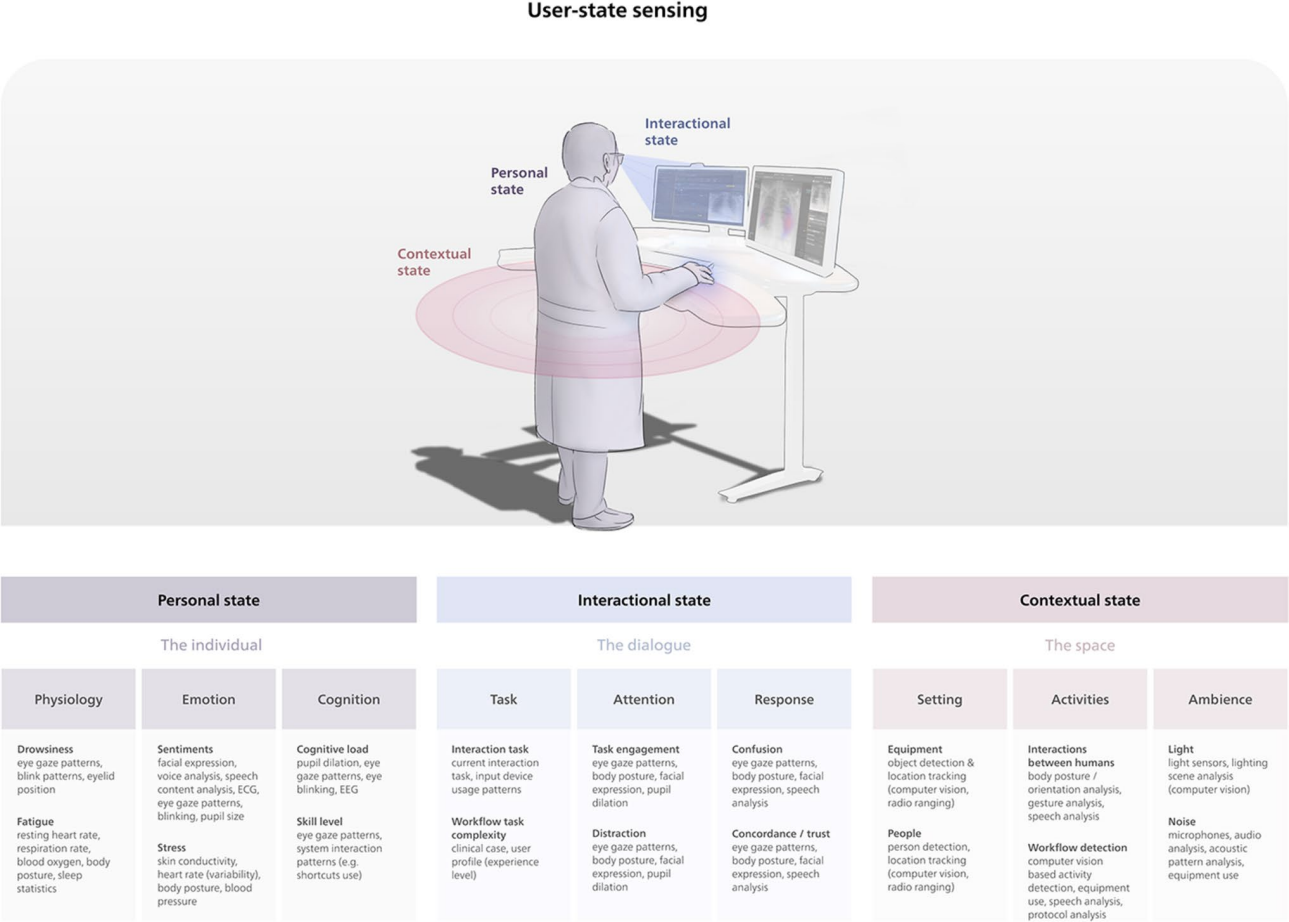
A schematic representation of the USSF. The user, their interactions, and the context these occur in are described in terms of a personal, interactional, and contextual state. Together these terms describe the complete user-technology interaction as it can be sensed by a system

User-state sensing is a combination of sensing and measurements of the user’s personal, interactional, and contextual states, as shown schematically in Fig. 1. This multifaceted approach incorporates biometric data, interaction dynamics, and environmental context to provide a comprehensive understanding of user-technology interaction.

### Lab Setup and Requirements

The Radiology AI lab serves as the initial physical implementation of the USSF, providing a simulated real-world radiology environment. The lab operates outside the regular clinical setting, with participants enrolled as research subjects to read non-clinical scans. This setup necessitates a physical space that is accessible to participants while remaining distinct from their usual clinical workspaces.

To enable real-time sensing within the USSF, we employ three non-invasive biometric measurement techniques: eye-tracking, heart rate monitoring, and facial expression analysis. These methods require data sources that can be integrated into a comprehensive analysis. In this initial iteration of the lab, we prioritise accessible, commercially available products that allow data analysis using third-party software or export raw data for further processing.

The personal and interactional states are continuously and non-invasively monitored through the selected biometric measurement techniques. User observations, think-aloud protocols [12], and detailed questionnaires further contribute insights into these states. The primary focus in this initial iteration is on capturing the personal and interactional states in real time, while incorporating the contextual state remains a focus for future work.

### Pilot Test

**Fig. 2:**
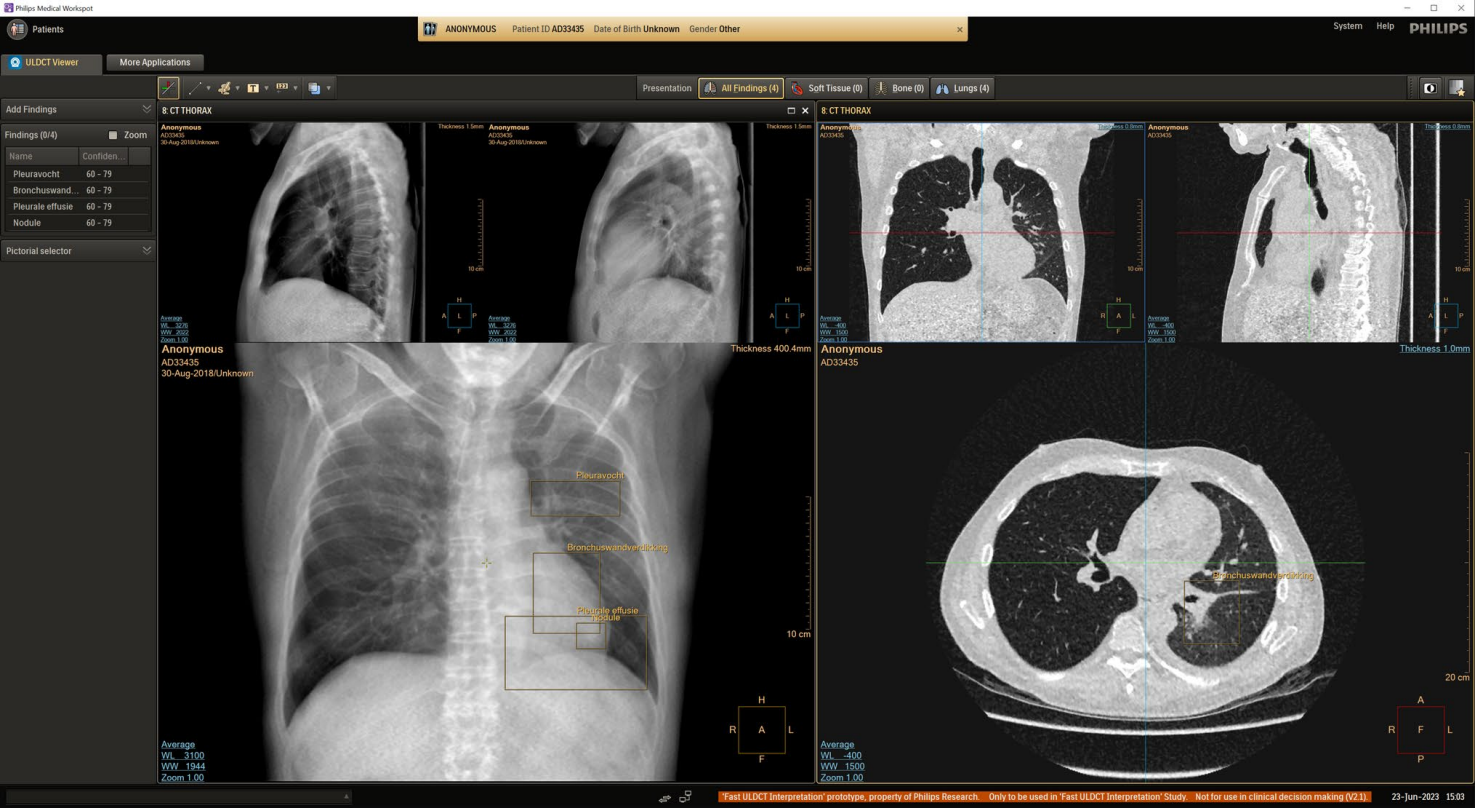
A screen capture of the PACS workflow that is enhanced with additional visualisations (shown on the left) and overlays of manually annotated findings. Hovering the mouse over the list of findings in the top left corner navigates to the location of the findings in the CT viewer on the right

To evaluate the lab setup and test the end-to-end data analysis of various biometric measurement devices, we conducted a pilot test comprising two goals. The primary analysis focused on comparing two applications and is not reported here. In the secondary analysis, we compared the biometric data of four radiologists with different experience levels as they read non-contrast ultra-low-dose CT (ULDCT) scans of the chest in both a standard, i.e. without AI features or assistance, picture archiving and communication system (PACS) environment, and a PACS environment that included additional visualisations and visual annotations of findings that were presented as though they were generated by AI. An example of how the visualisations and annotations were integrated into the UI is shown in Fig. 2. We aimed to identify relevant measurement axes for the lab and assess the feasibility of the setup. The PACS viewer was developed specifically for this study and included all functionality of a standard PACS.

The participants represented different levels of experience, with a resident with 4 years of training, a junior radiologist who completed a fellowship in thoracic imaging with 1 year of clinical practice experience, a thoracic imaging fellowship-trained senior radiologist with over 10 years of clinical practice experience, and a thoracic imaging fellowship-trained very senior radiologist with over 30 years of clinical practice experience.

The primary objectives were to confirm established trends in the literature, particularly the correlation between eye-tracking metrics and experience level [13], and to explore the potential relevance of additional biometric measurements, including heart rate monitoring and facial expression analysis. To achieve this, eye-tracking data was used to compute the duration and quantity of fixations and saccades, as well as track the diameter of the pupil of participants. Heart rate variability (HRV) will be computed using measured RR intervals. Facial expressions are quantified in terms of valence, which represents emotional states on a scale, where positive and negative emotions are expressed numerically. Finally, interpretation time is tracked between the two workflows. The measured biometrics are evaluated for statistical significance using one-sided *t*-tests at an alpha of 5%, to reflect the investigation into known trends in literature. 

Four radiologists participated in the pilot, each reading 32 ULDCT cases in both PACS environments. Participants completed four reading sessions, with 16 scans per session, conducted in either the morning or afternoon. The scans were presented in a randomised order to minimise potential bias from fatigue or sequence effects. Interpretation times were recorded, and various (bio)metrics related to their interaction with the software applications were computed. Throughout the sessions, at least two observers were present to monitor the participants.

## Results

**Fig. 3:**
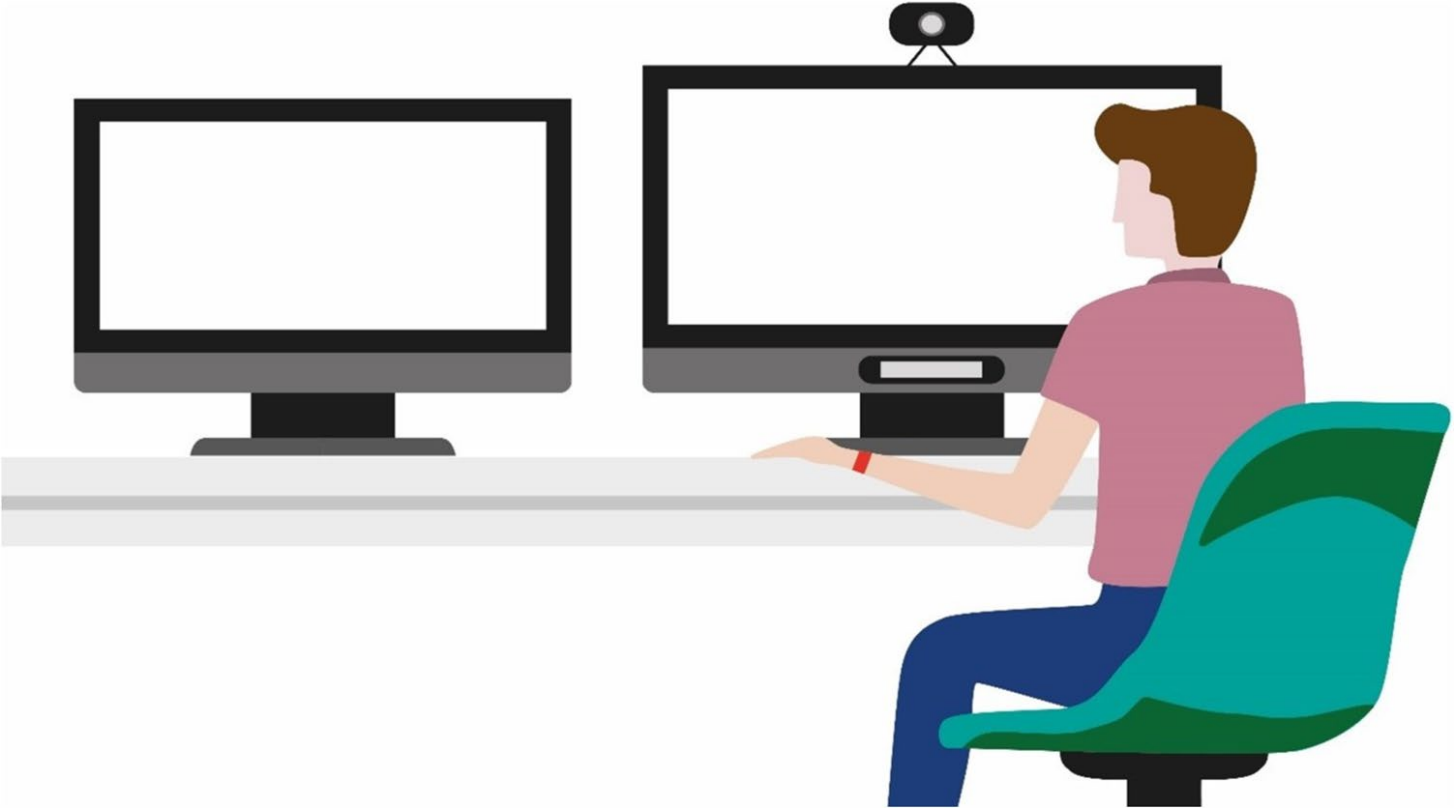
Schematic representation of the Radiology AI lab set-up. The primary monitor on the right is used for the primary software application, and the secondary monitor on the left is used to display patient information. The radiologist is wearing a heart rate monitoring wristband, and their eye movements are tracked on the primary monitor using an eye-tracker. A webcam captures facial expressions and serves as a microphone. All screens are recorded to capture events

The Radiology AI lab was realised in a dedicated room within the Leiden University Medical Centre, located near the main patient diagnostic areas. The lab was created through collaboration between radiologists, researchers, and scheduling teams, providing a simulated setting that replicates a standard clinical radiology workstation. The lab setup allows radiologists to participate in experiments with one or more observers present. The radiologist is seated at a desk equipped with two monitors, as shown schematically in Fig. 3. The left monitor displays patient information, while the primary monitor on the right is used for diagnostic imaging within a specific software application.

**Fig. 4:**
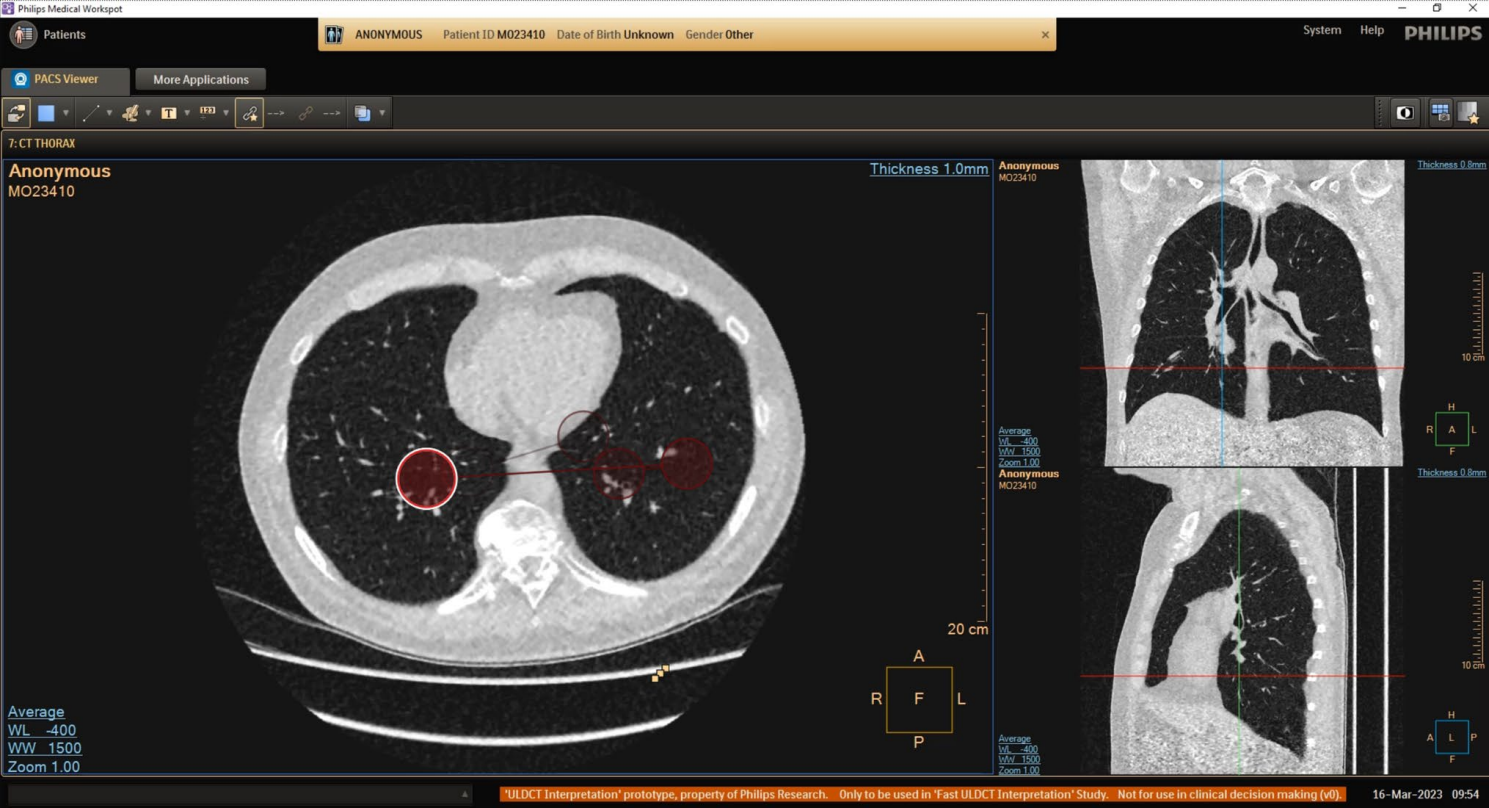
Example of raw output from the eye-tracking Tobii Pro Lab software. Gaze points, represented by red circles, indicate participant fixations. The size of the circle corresponds to the duration of the fixation at that point

To meet the lab’s requirements, as set out in the methods section, we utilise three biometrics measurement devices and record all three monitors. A Tobii Nano eye-tracker is mounted beneath the primary monitor to track gaze and capture various eye-related metrics. Tobii Pro Lab software integrates the eye-tracking data with audio and video streams from the webcam and additionally allows for reviewing of eye-tracking data alongside screen recordings, as illustrated in Fig. 4. The data is stored locally and can be exported in CSV format.

Radiologists wear a Corsano Cardiowatch 287-2 wristband, which records heart rate, skin tone, and temperature. This data is synchronised with the cloud via Bluetooth and can be exported in CSV format. A webcam mounted on the primary monitor captures facial expressions and serves as a microphone. The webcam recordings are analysed using py-feat, an open-source facial expression analysis toolbox, which computes valence scores and various emotional expression metrics from video stills. Additionally, a backup camera is placed behind the radiologist to capture audio and video during experiments, which is used to reference the proceedings during the pilot test. A detailed breakdown of the costs for the hardware and software used to construct this lab is provided in Table 1.

**Table 1:** Overview of estimated equipment costs for the Radiology AI lab. Prices are based on purchases made in 2022–2023. Furniture costs were not incurred as materials were provided in the hospital as standard equipment. PACS and reporting engine costs are reported as optional as these are present in the hospital as standard equipment

Equipment	Estimated cost
***Furniture***	€ 1.500
- Desk	
- Desk chair	
- Observer chair (2)	
***Computing hardware***	€ 4.000
- Workstation	
- 28-inch screen (2)	
- Mouse and keyboard	
***Hardware***	€ 500
- GoPro Hero 10 and mount	
***Software***	€ 11.000–16.000
- Tobii Pro Studio License	€ 3.000 € 5.000–10.000
- Viewer/PACS software (optional)	
- Reporting engine (optional)	€ 3.000
***Biometrics***	€ 2.500
- Tobii Nano Pro	
- Corsano Cardiowatch 287-2	
- Logitech Webcam	

### Pilot Test

All four participants read the 32 ULDCT cases (mean age, 52 years ± 23 (SD); 17 male; 16 cases with abnormalities) in both workflows in the pilot test. Cases were read on average in 4.1 ± 2.2 min (standard PACS) and 3.9 ± 1.9 min (AI-annotated PACS), with no significant difference (*p* = 0.48). Eye-tracking data was analysed to compute saccade duration, saccade quantity, fixation duration, fixation quantity, and pupil diameter. The results are presented in Table 2. Trends showed that saccade duration, saccade quantity, and fixation quantity increased with experience, while fixation duration and pupil diameter decreased with significant variance (ANOVA, *p* < 0.05). Excluding saccade quantity for the junior participant, all participants showed significant shifts (*p* < 0.02) aligning with the direction identified in literature, suggesting increased competency and reduced cognitive load in the AI-annotated workflow.

**Table 2:** Pilot test results from eye-tracking data, showing average saccade duration (*d.*) in ms, saccade quantity (*q.*), fixation duration (*d.*) in ms, fixation quantity (*q.*), and pupil diameter (diam.) in cm with standard deviation. Results are reported per participant and per workflow (W; 1 PACS, 2 AI-annotated PACS), with trends from the literature indicated. All metrics shifted significantly between the two workflows except for *

	W	Saccade *d*	Saccade *q*	Fixation *d*	Fixation *q*	Pupil diam
Resident	1	24.2 ± 1.8	412.5 ± 155.1	561.2 ± 107.7	298.1 ± 91.4	3.36 ± 0.10
	2	25.5 ± 1.6	579.6 ± 238.9	464.9 ± 87.4	422.5 ± 149.9	3.25 ± 0.09
Junior	1	31.6 ± 1.1	497.4 ± 153.6*	490.3 ± 60.7	380.4 ± 121.7	2.75 ± 0.11
	2	33.1 ± 1.6	554.5 ± 266.0*	446.9 ± 53.2	467.5 ± 226.0	2.69 ± 0.07
Senior	1	25.2 ± 2.5	614.2 ± 257.6	393.9 ± 47.0	427.3 ± 160.9	3.25 ± 0.21
	2	28.5 ± 3.0	748.9 ± 329.3	358.7 ± 46.3	534.8 ± 232.4	3.15 ± 0.16
Very Senior	1	27.2 ± 1.8	342.6 ± 120.2	383.0 ± 61.1	194.4 ± 56.9	3.16 ± 0.08
	2	27.2 ± 1.8	425.6 ± 213.7	331.5 ± 56.8	232.7 ± 90.0	3.10 ± 0.07
Trend in literature		Up [13]	Up [14]	Down [15]	Up [16]	Down [17]

**Fig. 5:**
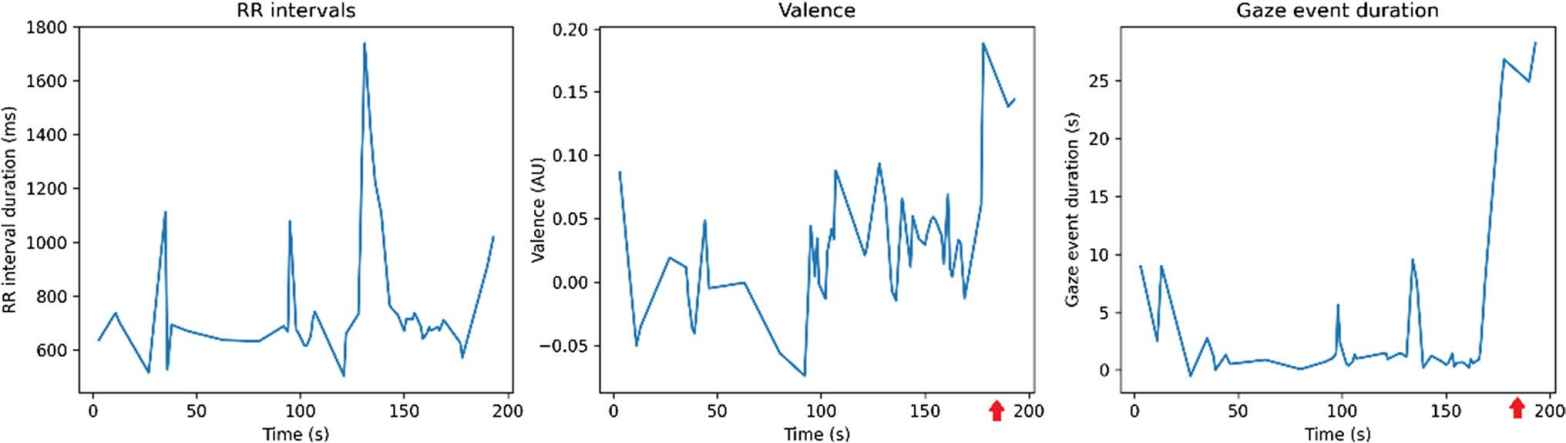
RR intervals, valence, and gaze event duration measured during the reading of a case. A significant increase in valence is observed around 185 s (denoted with an arrow), corresponding to the participant’s realisation that they had missed a finding. The increase in gaze event duration at this point reflects the participant looking away at the second monitor to correct their report. The peak in RR intervals at around 130 s likely corresponds with a missed beat-to-beat measurement

**Fig. 6:**
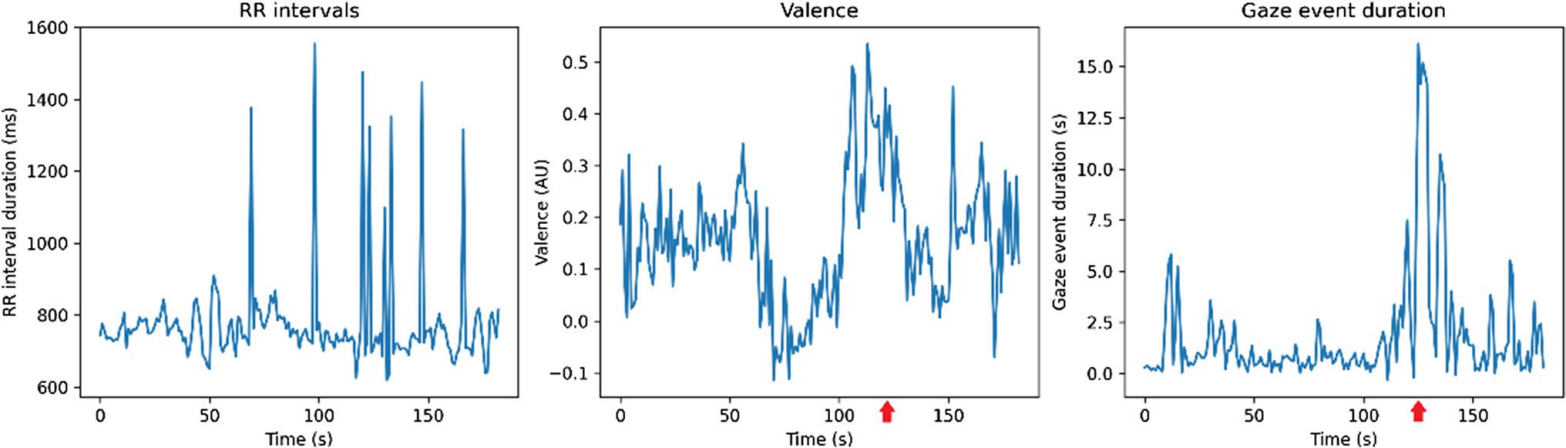
RR intervals, valence, and gaze event duration as measured during the reading of a case. A crash of the recording software happened around 120 s (denoted with an arrow), which is recorded as disturbances in the three measured values

Heart rate monitoring data was analysed to calculate mean RR intervals as a metric of HRV. In isolation, HRV metrics showed no significant results. Webcam footage was analysed to compute the facial valence, with measurements taken once per second. Two example instances where these measurements coincided with noteworthy events are included in Figs. 5 and 6. Figure 5 provides an example, where a participant reported missing a finding near the end of their case review. This coincided with a noticeable drop in facial valence. In Fig. 6, an example is included where the recording software crashed.

## Discussion

Several studies have investigated radiologists’ interactions with AI software, offering insights into their perspectives on AI and its impact on productivity [8, 9, 18]. These insights have primarily been gathered through surveys. The Radiology AI lab builds on this research by incorporating biometric measurements to provide objective data on the interaction between radiologists and software applications. The lab utilises affordable and commercially available devices providing access to raw measurement data to cover multiple dimensions of the proposed USSF.

The pilot test demonstrated the practicality of the Radiology AI lab and highlighted biometrics relevant to assessing user-technology interaction. In a direct comparison between workflows with and without AI, interpretation times did not differ significantly, but biometrics did. These evaluations lay the groundwork for future experiments that will further explore how AI impacts radiologists’ efficiency, decision-making, and workflow integration.

### Establishing the Radiology AI Lab

Locating the Radiology AI lab near clinical areas offers advantages in facilitating radiologists’ participation in experiments. However, securing appropriate space can be challenging, and the startup costs related to hardware acquisition are substantial. Success relies heavily on the availability and willingness of radiologists to participate, underscoring the importance of effective communication with institutional planning and coordination teams.

### Measuring Biometrics in the Radiology AI Lab

Eye-tracking has proven to be a valuable tool for capturing quantitative and objective data in various perceptive and cognitive research fields [16, 17], with significant applications in diagnostic radiology [19, 20]. Radiology, which heavily relies on visual information processing for clinical interpretation, provides key insights into the methods used by radiologists. Visual search patterns have been shown to correlate with a radiologist’s experience [13, 21] and level of comfort [19]. Analysing eye-tracking data through metrics such as fixation duration, the number of fixations, and saccades offers valuable information about mental load and task performance [19].

Cardiovascular measures also serve as reliable physiological indicators of mental load and user well-being [22]. Cardiovascular monitoring is easy to implement with minimal discomfort. HRV, which reflects the variability in beat-to-beat intervals, is a well-established metric that responds to changes in blood pressure and mental stress [23]. In the Radiology AI lab, HRV data can be used to identify when a radiologist is approaching their cognitive capacity, offering insights into workload management and subconscious preference for software applications.

Facial expression analysis complements eye-tracking and heart rate monitoring by providing an additional layer of assessment in user interactions with software. Facial expressions convey mental states and can offer real-time indications of a participant’s focus, commitment to task, and potential distractions or interruptions. One commonly used metric to quantify facial expressions is valence, which represents emotional states on a scale, where positive and negative emotions are expressed numerically [24]. Analysing valence can further our understanding of user engagement and performance during interactions with AI tools.

### Pilot Test

In the pilot test, eye-tracking metrics showed correlations with radiologists’ experience level, consistent with findings in previous studies [18, 20, 21, 24]. Additionally, a significant shift in eye-tracking metrics was observed when comparing a regular PACS workflow to an AI-annotated PACS workflow, providing insight into the subconscious interaction between the radiologist and the workflow. Though a speed-up in interpretation time was not found, these metrics suggest a benefit in user experience of using the AI-annotated workflow.

While differences in HRV metrics were observed between participants, these metrics, in isolation, did not provide additional insights beyond existing literature. HRV metrics computed for short durations (e.g. 5 min or fewer, as was the case in the pilot test) may not be directly comparable to established trends from longer-term HRV studies [25]. Similarly, facial expressions are primarily influenced in social contexts [26] limiting their value in isolated settings.

However, combined metrics proved valuable in identifying significant moments during the pilot test. As illustrated in Figs. 4 and 5, spikes or deviations in HRV, facial expression, or eye-tracking data often coincided with notable interruptions in the radiologist’s workflow. By integrating these measurements, we can pinpoint specific interactions between the radiologist and the software that warrant further examination.

### Expanding the Radiology AI Lab

The USSF guided the selection of the devices used in the Radiology AI lab. These devices were chosen for the accessibility, cost-effectiveness, and the relevance of their outputs, covering aspects of the personal and interactional states within the framework. Future expansions of the AI lab could enhance the representation of the contextual state by incorporating measurements of participants’ surroundings, e.g. in terms of light or noise in a room, an area left for future work.

The devices used in the set-up for the Radiology AI lab may play a larger role in future comparative studies. Eye-tracking can help quantify the interaction with potential AI features. The combined use of HRV metrics and facial expression analysis may provide additional insight into participant’s preferences during experiments.

One of the strengths of the Radiology AI lab is the ability to deploy the setup to live clinical settings, for example, by integrating eye-tracking and HRV monitoring into diagnostic workstations. This would allow for in situ evaluations of AI tools, capturing the impact on workflow efficiency and decision-making in real time.

### Limitations

The Radiology AI lab was established in a controlled, closed-off environment using a combination of commercially available and open-source software alongside a hardware configuration designed to emulate the institution’s standard clinical workstation. However, this setup may not be directly replicable in other institutions, presenting a potential limitation. For example, increasing the number of monitors to more closely reflect professional-grade workstations would necessitate additional eye-tracking devices, thereby increasing complexity and cost. Additionally, the choice of PACS and reporting software can significantly influence the baseline workflow efficiency, introducing variability that may impact study outcomes. Future comparative studies across different centres must account for these institutional differences to ensure valid and reliable assessments of radiologist-AI interactions.

While the pilot test provided detailed information about individual users, several challenges remain. One key challenge is the simulated lab environment. In our experiments, radiologists read CT scans but did not need to authorise reports, meaning no clinical decisions were at stake. Additionally, the lab setting shielded participants from typical workflow interruptions and colleague interactions. Participants noted the lack of typical interruptions in the lab environment, which may have reduced stress levels. Achieving greater realism in future experiments would help address gaps in the USSF. Finally, incorporating baseline measurements into experimental setups would enhance comparative studies by providing a frame of reference to measured biometrics, thus providing an indication of the impact of the AI application on users and their interactions.

## Conclusion

The Radiology AI lab was successfully realised, implementing personal, interactional, and contextual states of the USSF, enabling objective analysis of radiologists’ workflows, and effectively capturing relevant biometrics. Future work will focus on expanding sensing of the contextual state of the user-state sensing framework, refining baseline determination, and evaluating AI-enabled tools in radiology workflows.

## Data Availability

The data used in this study will not be made available publicly due to privacy concerns.
